# Medical Genetics, Genomics and Bioinformatics Aid in Understanding Molecular Mechanisms of Human Diseases

**DOI:** 10.3390/ijms22189962

**Published:** 2021-09-15

**Authors:** Yuriy L. Orlov, Anastasia A. Anashkina, Vadim V. Klimontov, Ancha V. Baranova

**Affiliations:** 1 The Digital Health Institute, I.M. Sechenov First Moscow State Medical University of the Ministry of Health of the Russian Federation (Sechenov University), 119991 Moscow, Russia; nastya@eimb.ru; 2 Agrarian and Technological Institute, Peoples’ Friendship University of Russia, 117198 Moscow, Russia; 3 Engelhardt Institute of Molecular Biology, Russian Academy of Sciences, 119991 Moscow, Russia; 4 Research Institute of Clinical and Experimental Lymphology—Branch of the Institute of Cytology and Genetics, Siberian Branch of Russian Academy of Sciences (RICEL—Branch of IC&G SB RAS), 630060 Novosibirsk, Russia; klimontov@mail.ru; 5 School of Systems Biology, George Mason University, Fairfax, VA 22030, USA; abaranov@gmu.edu; 6 Research Centre for Medical Genetics, 115522 Moscow, Russia

Molecular mechanisms of human disease progression often have complex genetic underpinnings, and sophisticated sequencing approaches coupled with advanced analytics. Modern computational approaches for the search and analysis of potential drug targets critically depend on the ability to reconstruct gene networks and to model the protein structure. New biomarkers derived from transcriptomics analysis or the standalone mining of associative networks aim to aid physicians to consider individual cases. This Special Issue continues the collection of papers on “Medical Genetics, Genomics and Bioinformatics” published in this journal in the wake of a series of medical research conferences held in Russia in 2020. The presented analytic techniques were discussed at the medical forum coordinated by I.M. Sechenov First Moscow State Medical University, and at the “Systems biology, bioinformatics and biomedicine” symposia held during BGRS-2020 biannual computational biology meeting in Novosibirsk, highlighting recent advances at the biomedical frontier. This collection of papers showcases insights in the field of human genomics, transcriptomics and proteomics, as well as some work conducted in model organisms. The current Special Issue contains eight research manuscripts and two reviews, each concerning some model or a pipeline applied to extract information useful for understanding molecular underpinning for a human disease and suggestive of a mechanism-specific treatment. We continue the previously published set of paper collections with an overarching theme of bioinformatics and medical genetics, which began in 2019 (https://www.mdpi.com/journal/ijms/special_issues/Medical_Genetics_Bioinformatics, accessed on 25 August 2021) [1]. This Special Issue is focused on the deciphering of molecular mechanisms underpinning common chronic diseases, including mental disorders, and emphasizing searches for drug targets. 

Computational models for systemic human disorders are now in high demand. Regulation of the so-called “normal” state of the cell is extremely complex. Gene expression may be controlled at transcriptional, post-transcriptional, and translational levels, and in the gene networks and pathway levels as well. This list is as endless as the research on this field will be. The disease models derived from this research are, however, very practical. New biomarkers derived from the transcriptomics analysis or standalone mining of associative networks aim to aid physicians to consider individual cases. The current series of post-conference journal Special Issues started with a coverage of Bioinformatics of Genome Regulation and Structure (BGRS) conferences and related Schools on Systems Biology and Bioinformatics (SBB) held in Novosibirsk, Russia [2,3,4,5,6]. This particular Special Issue contains the manuscripts that have followed on from oral and poster presentations discussed at the “Systems biology, bioinformatics and biomedicine” (SbioMed-2020) symposium in Novosibirsk, as well as the conferences at I.M. Sechenov First Moscow State Medical University in Moscow [6]. The majority of these papers present some insights into the molecular mechanisms of various human diseases, and their progression. 

We open this collection of papers with gene network modeling studies. The paper by Olga Saik and Vadim Klimontov describes a gene network related to glucose level variability in diabetes [7]. A growing body of evidence indicates that excessive glucose fluctuations serve as a risk factor for microvascular and macrovascular diabetic complications [8]. Accordingly, glucose variability is increasingly recognized as a therapeutic target [9]. Recent data indicate that deteriorative effects of glucose variability in the target organs are realized through the up- and down-regulation of a large set of genes [10]. In such cases, the analysis of gene networks can provide valuable information for a comprehensive understanding of disease pathogenesis. To reconstruct relevant networks in the automatic mode, authors employed the ANDSystem (Associative Network Discovery System), which is based on text mining, an automatic knowledge extraction from the texts of scientific publications [11,12,13]. The reconstructed gene network of glucose variability consists of 37 genes associated with both hyperglycemia and hypoglycemia. The identified genes are involved in insulin secretion, glucose homeostasis, as well as some signaling pathways which regulate cellular metabolism, cell cycle, and cell–cell interactions. Interestingly, the genes associated with glucose variability turned out to be hubs in gene networks describing diabetic vascular complications. A number of new candidate genes, promising for experimental verification of their role in glucose variability, have been identified as well.

Anna V. Glyakina and colleagues presented a spatial model of filamentous actin (F-actin) organization in eukaryotic cells [14]. First, they employed electron microscopy, limited proteolysis, mass spectrometry, X-ray diffraction, and structural modeling to show that the double helical molecules of filamentous actin are inconsistent with the observed ladder-like stacking of G-actin into F-actin, which is evident from the EM images. Therefore, a novel model of stacking actin monomers in filamentous actin is proposed, where actin monomers form one filament to make the F-actin core as inaccessible to the solvent as possible.

The topic of protein structure modeling is continued by Dmitry Karasev and co-authors [15]. They developed an approach for the fuzzy classification of protein sequences based on the ligand structural features to analyze ligand–protein interactions for new therapies. The current study extended the topic of the protein–ligand models published recently in the Special Issue “Medical Genetics, Genomics and Bioinformatics” [16]. The protein kinase family case demonstrated the effectiveness of the proposed technique.

Larisa Litvinova et al. [17] studied the secretory activity of mesenchymal stem cells upon an in vitro contact with calcium phosphate coatings, with an important biotechnological consequence. The manufacturing of specific biomaterial surfaces may directly induce osteogenic differentiation in cells. Cellular and molecular reactions of mesenchymal stem cells with the plastic with a double-sided calcium phosphate coating show that there are correlations between the mRNA expression levels for the selected genes and the secretion of cytokines and chemokines that may potentiate the differentiation of these cells into osteoblasts [18].

Evgeny A. Ermakov and co-authors [15] studied the biochemical underpinnings of schizophrenia and have linked this condition to immunity and inflammation via immunoglobulins hydrolyzing histones. Schizophrenia is known for its association with chronic low-grade inflammation. Extracellular histones and nucleosomes may trigger systemic inflammatory and toxic reactions. The authors presented the first evidence that polyclonal IgGs of patients with schizophrenia effectively hydrolyze five common types of histones. 

Immunohistochemistry research was continued by the work of Anastasiya V. Snezhkina and colleagues [19]. The authors studied succinate dehydrogenase (SDHx) genes in carotid paragangliomas, a type of rare neuroendocrine tumor. SDHB gene immunohistochemistry could be useful for the initial identification of patients potentially carrying SDHx mutations necessitating genetic testing. This work extends the previous study of paraganglioma performed with bioinformatics methods [20] and presented at the post-conference Special Issue on bioinformatics [21].

Olga Redina et al. [22] dissected the molecular mechanisms of behavior in laboratory animal models. The authors used RNA sequencing to identify gene expression changes in the ventral tegmental area of mouse brains in animals undergoing agonistic interactions (fighting), a known model for the study of excitation of brain neurons and the formation of social behavior patterns. The author revealed a set of differentially expressed genes playing a role in the maturation of dopaminergic neurons under the influence of social stress.

Molecular mechanisms of behavior disorders were analyzed in the study by Marco Ragusa and colleagues [23]. This group analyzed associations among the alteration of salivary miRNAs, saliva microbiome structure, and autistic spectrum disorder (ASD). When the relationship between brain functionality and saliva bacterial populations is perturbed by pathological conditions, ASD may result. The authors present a statistical association of both miRNAs and microbes with neuropsychological scores related to social interaction anomalies. This work continues the studies of autism predisposition genes previously described in an *IJMS* Special Issue [24].

The topic of possible mechanisms of childhood-onset neurodegenerative disorders was continued by Elena Shematorova and George Shpakovski [25], who reviewed molecular mechanisms promoting the juvenile form of neuronal ceroid lipofuscinoses (Batten disease). This malady is caused by mutations in the *CLN3* gene, which is highly conserved in the evolution of all mammalian species [26]. Detailed analysis of recent genomic and transcriptomic data indicated the presence of human-specific features of its expression.

The review by Simone Donati et al. concludes the Special Issue [27]. The authors discuss the potential role of microRNAs in multiple endocrine neoplasia type 1 syndrome, a rare inherited tumor disease, characterized by the development of multiple neuroendocrine tumors. Deregulation of certain miRNAs species has been associated with this syndrome. The potential roles of miRNAs as future non-invasive diagnostic and prognostic biomarkers is discussed. 

Thus, the current Special Issue on medical genomics shows that bioinformatics tools for the systems analysis of human diseases are in high demand, as could be seen from recently published post-conference papers [28,29]. The outputs of disease model analysis come in the form of sets of genes and protein markers which represent a particular interest to medical practitioners [30]. The guest editors are happy to announce that the next Special Issue topic at MDPI *IJMS* will be on medical genomics (https://www.mdpi.com/journal/ijms/special_issues/Medical_Genetics_2021, accessed on 25 August 2021).

Based on the readers’ interest in the topic, we are continuing to focus on the materials in this scientific field based on novel computational approaches, digital medicine technologies, networks and metabolic pathways analysis.
